# Allele phasing is critical to revealing a shared allopolyploid origin of *Medicago arborea* and *M. strasseri* (Fabaceae)

**DOI:** 10.1186/s12862-018-1127-z

**Published:** 2018-01-27

**Authors:** Jonna S. Eriksson, Filipe de Sousa, Yann J. K. Bertrand, Alexandre Antonelli, Bengt Oxelman, Bernard E. Pfeil

**Affiliations:** 1 Department of Biological and Environmental Sciences, University of Gothenburg, Box 461, 40530 Gothenburg, Sweden; 2 Gothenburg Global Biodiversity Centre, Box 461, SE-405 30 Göteborg, Sweden; 3 Gothenburg Botanical Garden, SE-41319 Göteborg, Sweden

**Keywords:** Hybridization, Autopolyploidy, Allopolyploidy, NGS, Allele phasing, Gene tree, Phylogeny, AlloppNET, Network, Woody, Legumes, *Medicago*

## Abstract

**Background:**

Whole genome duplication plays a central role in plant evolution. There are two main classes of polyploid formation: autopolyploids which arise within one species by doubling of similar homologous genomes; in contrast, allopolyploidy (hybrid polyploidy) arise via hybridization and subsequent doubling of nonhomologous (homoeologous) genomes. The distinction between polyploid origins can be made using gene phylogenies, if alleles from each genome can be correctly retrieved. We examined whether two closely related tetraploid Mediterranean shrubs (*Medicago arborea* and *M. strasseri*) have an allopolyploid origin – a question that has remained unsolved despite substantial previous research. We sequenced and analyzed ten low-copy nuclear genes from these and related species, phasing all alleles. To test the efficacy of allele phasing on the ability to recover the evolutionary origin of polyploids, we compared these results to analyses using unphased sequences.

**Results:**

In eight of the gene trees the alleles inferred from the tetraploids formed two clades, in a non-sister relationship. Each of these clades was more closely related to alleles sampled from other species of *Medicago*, a pattern typical of allopolyploids. However, we also observed that alleles from one of the remaining genes formed two clades that were sister to one another, as is expected for autopolyploids. Trees inferred from unphased sequences were very different, with the tetraploids often placed in poorly supported and different positions compared to results obtained using phased alleles.

**Conclusions:**

The complex phylogenetic history of *M. arborea* and *M. strasseri* is explained predominantly by shared allotetraploidy. We also observed that an increase in woodiness is correlated with polyploidy in this group of species and present a new possibility that woodiness could be a transgressive phenotype. Correctly phased homoeologues are likely to be critical for inferring the hybrid origin of allopolyploid species, when most genes retain more than one homoeologue. Ignoring homoeologous variation by merging the homoeologues can obscure the signal of hybrid polyploid origins and produce inaccurate results.

**Electronic supplementary material:**

The online version of this article (10.1186/s12862-018-1127-z) contains supplementary material, which is available to authorized users.

## Background

Polyploidization has played an important role in plant speciation in nearly all groups of vascular and non-vascular plants [1]. Speciation through polyploidy is likely to be the dominant mode of sympatric speciation in plants, as genome doubling will usually cause reproductive isolation from the parents [2]. Recent reports estimate that as many as ~ 15% of speciation events in angiosperms, and up to 31% in ferns, are accompanied by changes in ploidy level [3]. Despite the intense investigation of polyploidization in plant evolution, understanding the evolutionary origin and relationships of polyploid taxa remains a major challenge.

### Polyploid mode of origin

Using their mode of origin as defining criteria [4], two classes of polyploid origin at the ends of a continuum can be distinguished: allopolyploidy and autopolyploidy. Allopolyploidy (hybrid polyploidy) results in genomes from different species residing in the same organism, with a higher than diploid total genome complement. At any given genetic locus, an allotetraploid will possess two genomes, each with two alleles (one pair from each parent). In contrast, autopolyploids arise from genome doubling within one species (or even of one individual). In standard phylogenetic analyses based on a single gene sequences, the alleles at a given locus (one homoeologue) from an allopolyploid species would, in the absence of incomplete lineage sorting (ILS), be expected to branch as sister to the parental genome lineage they originated from. The alleles at the other homoeologous locus would branch as sister to the other parental lineage. However, in autopolyploids all four alleles might be expected to group together.

In allotetraploids, both loci derived from the parental genomes typically remain distinct after genome merging. This means that a complete sample of alleles can allow the phylogeny of one genetic region (i.e., including two homoeologous loci from an allotetraploid) to show evidence for the two parental origins. However, obtaining this kind of data has been rather laborious up until now (e.g., using Sanger sequencing). In contrast, using next-generation sequencing (NGS), the generated reads will potentially contain enough information such that it should be possible to distinguish homoeologues and their allelic variants from one another. This is highly advantageous when it comes to inferring each parental lineage. Although, it is well-appreciated that individual gene trees may not match the species/genome tree for various reasons [5]. In particular, if speciation has been rapid, then incongruence due to the coalescent process [6, 7] needs to be taken into account, which in turn calls for the sampling of several unlinked loci [8, 9]. Such an enlarged sample will also mitigate the effects of missing or unrecovered alleles/loci in individual gene trees. Sampling many loci is especially important to avoid being misled by seeing one pattern among gene trees (conforming to the expectations of one mode of polyploidy), when only a few genes have been sampled, due to stochastic factors.

In typical allopolyploidy, only the two homologous chromosomes from the same parental species pair at meiosis (thereby forming bivalents) and consequently display disomic segregation [10]. This means that meiotic recombination only occurs between those chromosomes contributed by each parental species, i.e., only within the parental genome, rather than between them. This in turn allows for the independent divergence of the alleles at each homoeologous locus, as well as the maintenance of differences inherited from the parents. In contrast, in recent autopolyploids the high similarity between chromosomes may lead to multivalent formation (more than two chromosomes form complex ‘pairs’ during meiosis, allowing recombination), or bivalent formation but with a new partner in each generation. Either of which enables polysomic segregation [11], i.e., all four alleles participate in the same recombination pool over many generations. This type of segregation makes it possible for an allele from only one parent to become fixed at a given locus. If disomy is subsequently re-established, divergence of the alleles at each locus can proceed, but only from the time that the ancestral allele became fixed.

In addition to these inheritance behaviors describing typical cases of genome duplication at each end of the polyploidy spectrum, intermediate cases can integrate features from both classes. The extent of this will depend largely on the degree of divergence (at the structural and sequence levels) between the parental genomes. Loci in allopolyploids may not remain distinct when certain kinds of chromosomal pairing occur. Some allopolyploids show multivalent formation (and therefore polysomic segregation) in only a restricted part of the genome. The rest of the genome forms bivalents without polysomic segregation [12, 13]. This is called segmental allopolyploidy and is thought to usually be a temporary state until disomy takes over, if there were sufficient differences among the parental chromosomes to favor the complete elimination of multivalent formation [13]. Thus, there may only be a short window during which cytological tools are useful to detect some kinds of segmental allopolyploids, namely those that have completed the transition to disomy. The phylogenetic signature of this mode of polyploidy is expected to include some homoeologous loci that diverge when (or earlier than) the parental lineages diverged, and some loci whose allelic variation is reset via polysomic segregation and drift, and instead show divergences that track the onset of disomy [14].

### Using NGS data to determine Polyploid mode of origin

Low-copy nuclear genes are particularly useful to infer the history and origins of polyploid taxa. These genes typically retain information about the reticulate history of hybrids via the gene copies received from each parent [15, 16]. Sampling many such genes has become cost efficient through gene capture techniques coupled with next-generation sequencing (NGS), where selected genes positioned throughout the genome can be targeted [17, 18]. Recent projects have started deciphering the complex genomes of polyploids, utilizing short read high-throughput sequencing for constructing haplotypes within known polyploids and their diploid parents [19–22]. However, a challenge remains when using short read data to assemble and phase all alleles/loci in polyploids. Phasing here refers to the segregation and assembly of sequence reads corresponding to different alleles in heterozygous loci. Specific tools for phasing polyploids have not yet been developed, and the problem increases in severity as more alleles per locus are present in a genome. The lack of a reference sequence (either haploid or diploid) from a close relative further compounds the problem [23], as does insufficient read depth [24]. Some studies avoid these issues by using a single consensus sequence to summarize all alleles at a locus for downstream phylogenetic analyses [25–27]. This approach could create chimeric sequences that may interfere with species tree reconstruction, conceal signals of polyploidy, and make it impossible to infer their mode of origin.

### Tetraploids in Medicago

The plant genus *Medicago* L. (Fabaceae) has undergone several polyploidization events in the wild and through cultivation. One such example is alfalfa (*M. sativa* subsp. *sativa* L., hereafter *M. sativa*), which is the most widely cultivated forage legume in the world with a production covering approximately 32 million hectares [28]. *Medicago arborea* L. and *M. strasseri* Greuter, Matthäs & Risse are closely related tetraploid species (2*n* = 4*×* = 32) with uncertain origins [28, 29]. Their evolutionary history is of particular interest, because they (along with the remaining member of section *Dendrotelis* not sampled here, *M. citrina* (Font Quer) Greuter) are the only species with hard woody stems that form shrubs, in contrast to herbaceous habits found in the other species of *Medicago* and the most closely-related genera *Melilotus* L. and *Trigonella* L. This opens up the possibility that a polyploid origin was directly coupled to the origin of the woody shrubby habit. In general, woody plants are not associated with high rates of polyploidy [1], so further information about specific cases is needed. Woodiness is also thought to be ancestral to an herbaceous habit in *Medicago* [30]. Rosato et al. [29] used cytological methods to study these two *Medicago* tetraploids, along with a number of other species, but could not resolve whether they have an auto- or allotetraploid origin. This remains an unanswered question that we tackled with a phylogenetic approach.

### Aims

In this study we develop a new analytical framework that uses NGS data from low-copy nuclear genes in order to reveal the complex evolutionary history of polyploid taxa. We apply this framework to 1) separate homoeologous sequences for each locus and phase their respective alleles; 2) compare the species tree/network using our method with the tree inferred from the same loci where the alleles’ majority consensus sequences were used instead of phased alleles; 3) test if *M. arborea* and *M. strasseri* arose from an auto- or allotetraploid event; and 4) examine whether these two species share a single polyploid origin, and whether this origin is correlated with their shared woody and arborescent habit.

## Methods

### Sampling and DNA extraction

We sampled three individuals of *Medicago arborea* L. and one of *M. strasseri* Greuter, Matthäs & Risse, and species from the *M. sativa* group and from sections *Lupularia, Platycarpae* and *Spirocarpos*, all indicated as close relatives of *M. arborea* in previous studies [31–33], for a total of 27 individuals (Additional file 1: Table S1). Most seeds were obtained from the USDA National Plant Germplasm System and were grown in growth chambers at the University of Gothenburg. Leaf samples and vouchers were obtained from each plant. DNA was extracted from silica dried leaf tissue using the DNeasy Plant Mini Kit (Qiagen, Valencia, CA, USA), following the manufacturer’s protocol.

### Gene selection, probe design and library construction

We used ten unlinked single copy nuclear genes previously selected from the reference genome of *Medicago truncatula* L. [34] and tested as phylogenetic markers in *Medicago* [17]. Library preparation and sequence capture was as per Sousa et al. [17]. In brief, genomic DNA was sheared with a Covaris S220 instrument (Covaris, Woburn, Massachusetts, USA) and DNA libraries were constructed using the NEXTflex DNA Sequencing Kit and NEXTflex Barcodes (BIOO Scientific, Austin, Texas, U.S.A.) together with Agencourt AMPure XP magnetic beads (Beckman Coulter) for fragment size selection and DNA purification. The MYBaits target enrichment system (MYcroarray, Ann Arbor, Michigan) was used for sequence capture of selected loci. Sequencing of the enriched DNA pools was done on a MiSeq platform from Illumina (San Diego, California, USA) at the Genomics Core Facility of the University of Gothenburg, Sweden.

### Contig assembly, allele phasing, alignment, recombination test

High-throughput 150 bp paired-end reads were processed using CLC Assembly Cell v.4.0.13 software (CLC Bio, Aarhus, Denmark). Adapter and quality trimming, with the default setting (threshold of 20 for the Phred-score), and a de novo assembly was performed for each sample to obtain contigs at each locus. Contigs corresponding to target loci were retrieved by creating a BLAST database for each assembly and running a query against the reference sequences in *M. truncatula*, using an E-value ≤1E-100*.* All target contigs were then aligned to the reference sequences using MAFFT v7.123 [35]. In each alignment, overlapping contigs belonging to the same species that failed to assemble into a single contig were manually merged to obtain longer sequences. With CLC-mapper, we used these sequences as a new reference for each locus, to allow more reads to be re-mapped to the corresponding sample.

Allele phasing was performed on the BAM files derived from read mapping using SAMTools phase [36], with default settings. In short, SAMTools calls heterozygous SNPs at one site and segregates the reads (which contain one or the other heterozygous SNP) into two new “phased” BAM files. Reads lacking the given SNP site (but in part overlapping the segregated reads) are segregated randomly to either BAM file. Given that SAMTools assumes site independency, all polymorphic sites (those not occurring on the same read or on the shared paired-end reads) will be treated as independent. This can result in switching errors, i.e., where polymorphisms belonging to one allele get allocated into the other allele [37, 38]. To correct for this kind of error we tested our phased alleles using recombination detection (see below). Switching errors are expected to decrease as SNP density increases, because most reads will contain more than one SNP, which should increase the correct segregation of reads into the phased BAM files.

Since the phasing procedure performed through SAMTools only assumes diploid species [37], allelic variants from tetraploid individuals had to be manually retrieved from the phased SAM files using TABLET [39] and Geneious v5.6 [40]. When more than two alleles were present, there would be additional polymorphisms in each SAM file that could be scored manually. For each tetraploid individual, we duplicated the two phased FASTA sequences in Geneious, to produce four different alleles that we modified by hand. The changes in the duplicated FASTA files were made by comparing both SAM files in TABLET and scoring unique polymorphisms that were not seen in either allele and that occurred in more than three independent reads (i.e., not three identical reads that may result from PCR duplication). The final sequences were aligned using MAFFT v7.123 and checked by hand.

Sequences were tested for recombination using RDP v.4.39 [41]. We used a *p*-value of 0.1 and three methods (RDP [42], MaxChi [43], Chimaera [44]) to initially screen for recombination events. Any putative recombination event was then re-checked with all methods using a p-value of 0.01 (GENECONV [45], BootScan [46], SiScan [47], 3Seq [48] and LARD [49]). The alignments were trimmed of all regions positively affected by recombination before the phylogenetic analysis.

### Phylogenetic analysis

For the phylogenetic analysis of individual genes, we used the reverse model jumping Markov chain Monte Carlo (rjMCMC) method, implemented in MrBayes v. 3.2 [50] to determine which substitution model was most visited during the rjMCMC search. We also included an among site rate heterogeneity parameter (gamma) for all models and genes, allowing rates to change across sites. This parameter is very commonly preferred by model selection methods [51] and we expected among site rate variation because of differences in evolutionary rates between exon and intron regions of each locus. We ran the Bayesian analysis with two parallel chains each for two independent runs of three million generations. We sampled every 1000 generations and accepted a burn-in of 10% after examination of the parameter convergence in Tracer v. 1.6 [52]. The phylogenetic analysis was carried out twice: first with gene alignments of phased alleles and secondly with alignments constructed using the majority consensus of the unphased reads for each individual that resulted in one sequence per sample.

BEAST v. 1.8 [52] analyses were performed for dating the nodes in our gene trees. Each alignment was subjected to one analysis with a strict clock and a second analysis with an uncorrelated lognormal relaxed clock model. The substitution model prior was selected from the most visited model in the MrBayes analysis (the rjMCMC analysis). The tree prior used was a Yule birth-death, with an estimated starting tree generated by the unweighted pair group method with arithmetic mean (UPGMA). We set a prior probability on the substitution rate (ucld.mean) using a normal distribution with a mean of 3.611E-9 and SD = 1.357E-9 substitution/site/year, based on earlier estimates of substitution rates in a set of low-copy nuclear genes that included the genes we selected here (Sousa et al. 2014). Monte Carlo Markov chains (MCMC) were run for 30 million generations, sampling the parameters every 6.000 generations. We used Tracer v.1.6 to check that the effective sample size (ESS) was > 200 for all parameters and that the runs had converged. Trees were annotated using TreeAnnotator (implemented in the BEAST package) after discarding 10% as burn-in to produce the maximum clade credibility tree with a posterior probability limit of 0.95. The final trees were visualized using FigTree v.1.4.2 (http://tree.bio.ed.ac.uk/software/figtree/).

The gene trees were time calibrated with two secondary calibration points, drawn from a reanalysis of matK data [53] depending on the clades retrieved from the gene trees in MrBayes. The two calibrations are the divergence between the *M. truncatula* and *M. sativa* clades and the crown age of *Medicago*. We modeled the divergence priors using a normal distribution with a mean of 6.14 Mya and standard deviation of 1.2, or a mean of 11 Mya and SD = 2.1, respectively, based on the results of Sousa et al. [54].

### Population size estimation

We estimated allelic diversity (*θ*_*w*_ [55]) for *Medicago arborea* by analyzing sequence polymorphisms in DnaSP v5 [56]. For each of the eight genes with two distinct clades of alleles from both *M. arborea* and *M. strasseri* (see Results), we used the alleles of *M. arborea* and calculated *θ*_*w*_ for each clade. The average number of alleles per estimate was 4.75. Coupled with locus-specific mutation rates (*μ*) [17], we produced 16 estimates of the effective population size. Some estimates could not be calculated, as *θ*_*w*_ was zero. For these entries we used instead the lowest overall *θ*_*w*_ across all estimations as a substitute when calculating the mean *θ*_*w*_. This is because the effective population size cannot realistically be zero (a non-zero result was returned by the other clade for the same locus and individuals in each case) and is probably due to stochasticity associated with observing polymorphisms in a low diversity sample.

### Distinguishing between hybridization and ILS

To test if the genes are affected by hybridization alone and not ILS, we used two approaches. Firstly, AlloppNET [57] implemented in BEAST v.1.8.1 [52], which uses the *BEAST model [58] and treats the diploid genomes of an allotetraploid as separate “species” (in the sense of the *BEAST model) with a shared species tree topology and population size after the hybridization event. In AlloppNET, diploid individuals have one “allele”, and tetraploids have two homoeologous “alleles” that are assigned to the correct genome using a stochastic parameter. In those genes where we have evidence of four alleles in our samples of tetraploid *M. arborea* and *M. strasseri* (eight of 10 genes), we defined two “individuals” (in the AllopNET sense) per sample (e.g. *arborea1* into *arborea1_1* and *arborea1_2*) and assigned one homoeologous allele per homoeologue to each “individual”. We used an R script provided by Graham Jones at his website (http://www.indriid.com/) to generate the BEAST xml file. This file was then edited to change the clock model to use a relaxed lognormal clock and then the MCMC was run for 300 million generations.

AlloppNet’s low support for several clades casts doubt on the finding of hybridization forming the polyploid taxa (see Results). We see two probable causes for the low support. Firstly, polyploidy may have occurred without hybridization. In this case, extensive ILS would be the reason for the observed non-sister placements of the two *M. arborea*/*strasseri* clades in most gene trees. We would expect these clades to be sister in the AlloppNet tree as well, but poorly resolved non-sister placements do not rule that out. Secondly, hybridization among the *diploid* lineages would violate an important assumption made in AlloppNet and could affect the inference of a real polyploid hybrid history, resulting in the poorly supported results we observed. We favor the second explanation because of supported contradictory trees seen using only the diploid taxa (Blanco-Pastor and Pfeil, unpublished results).

In order to further discriminate among these possibilities we applied the second test of a hybrid signal in phylogenetic trees [33, 59, 60]. This test was used to determine whether ILS or hybridization was the likely cause of the non-sister position of genomes from the tetraploid species in some of the gene trees. Although this test was designed to compare different gene trees from diploid taxa, with respect to the position of taxa among the trees [33], we adapted it to assess the differences in the phylogenetic pattern between a pair of homoeologous clades in polyploids, similar to what was done by Eriksson et al. [24]. In order to proceed with the test, we alternatively trimmed alleles from one of the homoeologous clades in each gene tree (leaving the other homoeologous clade) while retaining all diploid alleles. These resulting pairs of test trees were then compared to one another. This means that the test trees were identical except in regard to the tetraploid taxa, thus rejection of the ILS null hypothesis could only be due to the alternative positions of the homoeologues.

A null expectation for this test was generated via coalescent simulation (ILS only, using a population size of 204,000 gene copies [see Results] for all branches), treating each test tree as though it was a species tree, and determining if the comparison between test trees resulted in a greater difference between them than that expected under the null [33]. We used a BEAST-generated ultrametric gene tree (the trees used for time-calibration, above) with branch lengths in appropriate time units as input into the simulations, as has been done in the most recent iteration of this test [60].

## Results

The ten phylogenetic markers have a mean alignment length of 2572 bp (Additional file 2: Table S2), and are deposited in Dryad (10.5061/dryad.rf500). Across all ten markers the mean read depth (coverage) before allele phasing ranges between 14.9-386.6 reads. The tetraploid *M. arborea* and *M. strasseri* had a mean coverage between 52.7-167 reads (see Additional file 3: Table S3 for more detail of mean read depth and standard deviation for all accessions and markers).

### Allele phasing

We managed to separate at least two allelic copies from all individuals in our study, using the paired-end read information from Illumina sequencing and SAMTools phase. In the tetraploids *M. arborea* and *M. strasseri*, we can expect to find up to four alleles (homoeologues and their allelic variants), depending on the degree of homozygosity at each locus. In order to distinguish between genuine polymorphisms and sequencing error, we accepted an allelic variant only if a polymorphic site was supported by at least three reads. When the read depth was low, such as in intron regions, we observed that the SAMTools phasing step did not always result in single contigs covering each locus after read assembly. Using Geneious v5.6 [40], we manually joined non-overlapping contigs or partially overlapping contigs where no differences were present in the overlap, to produce larger fragments from remaining contigs after the SAMTools phasing step (overlapping but different contigs were kept separate). This could accidentally result in the merging of the 5′ (front) part of one allele with the 3′ (back) part of another, giving a chimeric fragment, especially if the area of overlap was in a region of low sequence variability. Although recombination within a genome (i.e., within a homoeologue) should not unduly affect a multispecies coalescent-based analysis [61], we expect that it could have a pronounced effect if alleles were recombined (in vivo or in silico) between homoeologues (i.e., containing sequences of different parental origins). We tested for recombination (using RDP4) in order to control for these possibilities. Putative in silico recombination was handled by swapping the ends of recombined fragments with one another when the breakpoints were shared and corresponded to the original contig boundaries. We removed (and replaced with Ns) the recombined parts of fragments in all other cases of detected recombination.

### Ploidy of M. strasseri

Our sample of *M. strasseri* was interpreted as tetraploid based on two factors. Firstly, the number of alleles observed in this specimen across loci (two to four per locus) was essentially the same as those observed in *M. arborea* (Additional file 4: Figure S1, Additional file 5: Figure S2, Additional file 6: Figure S3, Additional file 7: Figure S4 and Additional file 8: Figure S5). Secondly, the ploidy of two of the *M. arborea* samples used here was previously confirmed as tetraploid, based on chromosome counts [23] (Table S1). We recovered only one or two alleles per individual from confirmed diploid samples, as expected (Additional file 1: Table S1, Additional file 4: Figure S1, Additional file 5: Figure S2, Additional file 6: Figure S3, Additional file 7: Figure S4 and Additional file 8: Figure S5).

### Phylogenetic analysis

**Fig. 1:**
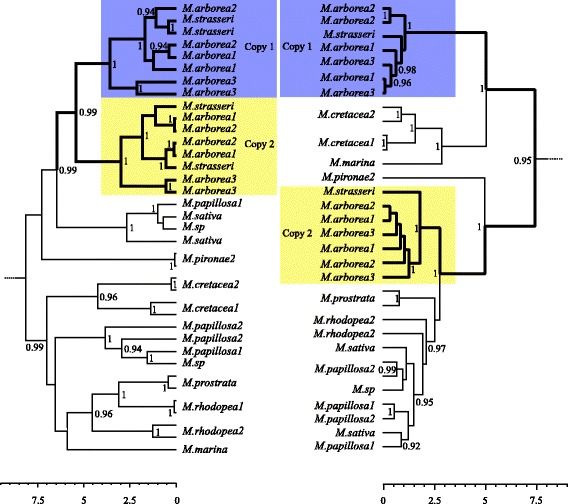
Gene trees matching an autopolyploid (left) and allopolyploid (right) mode of origin in two low-copy nuclear genes 1 and 9, respectively. Only Bayesian clade posterior probabilities above 0.9 are shown. The scale bar is in units of millions of years. Individuals (species and sample number within that species) included in the truncated clades Sativa, Truncatula, and Rotata, are as follows; *M. papillosa1, M. papillosa2, M. sp., M. rhodopea1, M. rhodopea2, M. prostrata and M. sativa: M. littoralis, M. italica, and M. truncatula: M. shepardii and M. rotata,* respectively. The blue and yellow *M. arborea* & *M. strasseri* clades contain what we interpret as one homoeologue each and their allelic variants

All Bayesian analyses had effective sample sizes above 200 for all parameters, indicating that the runs had converged to the posterior distribution. The most visited substitution model using a reverse model jump was HKY + G [62] in all genes. The phylogenetic inferences identified two principal well-supported topologies for the positions of the *M. arborea* and *M. strasseri* sequences. They either all together formed a clade (in gene 1, Fig. 1a) or formed two separate clades wherein each clade contained alleles from both species (e.g., in gene 9, Fig. 1b).

The first pattern was obtained in a single gene tree, where all *M. arborea* alleles formed a monophyletic group with all *M. strasseri* alleles, but with a substructure consisting of subclades, each containing *M. arborea* + *M. strasseri* alleles (Fig. 1a and Additional file 4: Figure S1). The second pattern, observed in eight of the remaining markers (i.e., apart from gene 3, see next), also displayed *M. arborea* alleles together with *M. strasseri* alleles, but this time the two monophyletic groups were well separated with diploids alleles branching between. One of these groups is usually closely related to a clade that includes *M. sativa*, whereas the other is placed elsewhere in the tree, e.g., sister to either *M. marina* or *M. pironae2* (Additional file 4: Figure S1, Additional file 5: Figure S2, Additional file 6: Figure S3, Additional file 7: Figure S4 and Additional file 8: Figure S5), with much variation observed between the trees at the fine scale.

The first pattern (in gene 1) might be due to tetrasomic segregation that has fixed the alleles from one parent, with the subsequent establishment of disomy allowing further divergence between these now independently evolving loci. The second pattern (in eight genes) is most likely explained by the presence of alleles from two homoeologues in an ancestor shared by *M. arborea* and *M. strasseri*. This is a pattern expected of taxa with an allopolyploid origin [63, 64]. Given the predominance of the second pattern, allopolyploidy appears to be the most feasible explanation of the variation seen within loci in these woody species of *Medicago*.

Finally, instead of only the *M. arborea + M. strasseri* grouping, consistent with a shared origin*,* gene 3 also retrieved a sister group relationship between some alleles of *M. strasseri* alone and *M. prostrata.* (Additional file 5: Figure S2). Fixation in this locus for *M. strasseri* is incomplete, indicating that this locus also resides in a part of the genome that may have been subject to tetrasomic segregation. The unusual pairing of *M. strasseri* and *M. prostrata* is congruent with diploid hybridization occurring in the common ancestor of *M. arborea* and *M. strasseri* prior to the allopolyploidization event, where sequences from more than one source lineage were transmitted to this common ancestor. This interpretation is further reinforced by the lack of a consistent topology among the diploid relatives of these tetraploids across loci seen here and previously [31, 33, 53, 54], especially in the *Bcop* locus, where *M. arborea + M. prostrata* was previously observed (*M. strasseri* not sampled) [33]. However, this needs to be further investigated.

Although the pattern typical for allopolyploidy (non-sister clades representing each homoeologue, [64]) was prevalent among individual gene trees built from phased alleles, it was almost entirely obscured when we used consensus sequences obtained from the majority nucleotide at each polymorphic site. We observed changes in supported relationships in five genes, involving taxa found close to either of the arborea/strasseri clades (red boxes in genes 2 – 4, 7, 9, Additional file 4: Figure S1, Additional file 5: Figure S2, Additional file 7: Figure S4 and Additional file 8: Figure S5). We also saw several reductions in support for formerly highly supported clades in seven of eight genes with separate clades of tetraploid alleles (genes 2, 4 – 9, Additional file 4: Figure S1, Additional file 5: Figure S2, Additional file 6: Figure S3, Additional file 7: Figure S4 and Additional file 8: Figure S5). This in turn masked the earlier inferences of either parental origin of the tetraploids. In gene 10 the relationships changed little, but in this case the two clades of alleles in the tetraploids were only separated by one weakly supported node (Additional file 8: Figure S5).

### Population size estimation

The median of 16 estimates of the effective population size was c. 147,000 gene copies (i.e., c. 73,500 individuals), the mean c. 204,000 and the distribution was left skewed (1st quartile c. 74,000, 3rd quartile c. 321,000). Excluding a single outlier (c. 596,000) returned median /mean / 1st quartile / 3rd quartile estimates of c. 146,000, 178,000, 62,000 and 301,000 gene copies, respectively (all values reported with three significant figures). We used the original mean as our point estimate of the population size (below).

### Distinguishing between hybridization and ILS

**Fig. 2:**
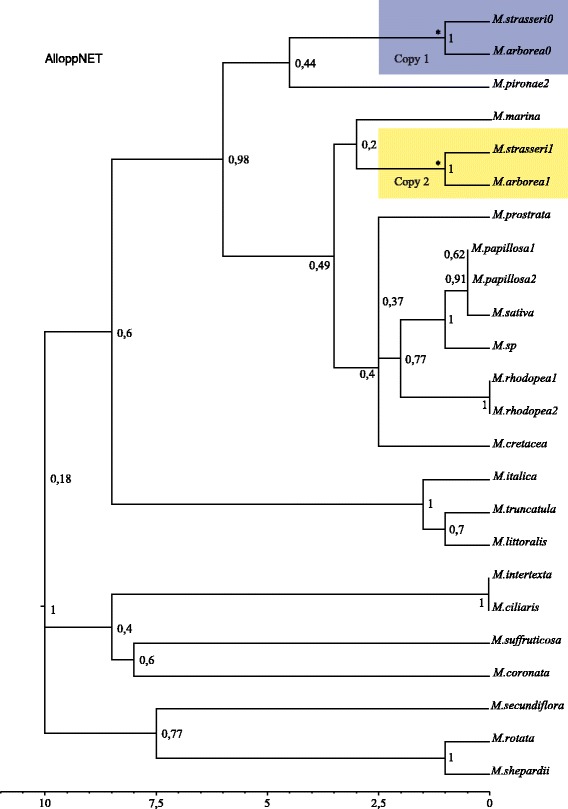
Species tree inferred using the AlloppNet model showing two parental lineages from which *M. arborea* and *M. strasseri* might have been derived. Clade posterior probabilities are shown next to the relevant branch

Although the pattern reported in the majority of gene trees is at first glance consistent with an allopolyploid origin, it is possible that incomplete lineage sorting could produce many topologies lacking a sister- relationship for homoeologues. This might occur if autopolyploidy was in fact the origin of these tetraploid species, but with disomic segregation established before most loci had a chance to become fixed for a single type of allele, with deeply coalescing alleles present at many loci. We tested this in AlloppNet [57] and recovered a single allopolyploid event shared by *M. arborea* + *M. strasseri*, with two possible parents, *M. marina* and *M. pironae2*, as responsible for the hybridization event, however the relevant clade posterior probabilities were below 0.5 (Fig. 2).

When we further tested for a signal of hybridization in each of the eight loci showing a non-sister pattern among tetraploid genomes (using a coalescent test based on gene trees), we found that the two positions of *M. arborea* alleles (corresponding to homoeologous genomes) resulted in trees that were significantly dissimilar compared to an ILS null in six of eight genes (Additional file 9: Table S4). ILS was rejected as the cause of the non-sister pattern of genomes in these cases.

## Discussion

### *Medicago arborea* and M. strasseri share an allotetraploid origin

Chromosome counts and different phylogenetic patterns shared by several loci present strong evidence that *M. arborea* and *M. strasseri* arose through allopolyploidization. Firstly, these species are clearly tetraploids: We have confirmed tetraploid chromosome counts in *M. arborea* [24] and although we did not count our *M. strasseri* specimen, the species has been reported to be tetraploid previously [28]. Further, the direct examination of sequence reads confirmed the presence of more than two alleles per locus. This visual approach is expected to suit other tetraploid species, where the correspondence between expected allele number and ploidy level can be used to check ploidy, e.g., Eriksson et al. [24].

We identified two subclades grouping *M. arborea* and *M. strasseri* that each includes one or two alleles from each *M. arborea* individual grouped with one or two alleles from the *M. strasseri* individual. These subclades were observed in 9 out of 10 gene trees. These two divergent subclades were usually not sister to one another (in 8 out of 10 gene trees) and in the majority of cases were also separated by several well supported nodes. We were able to reject in several genes the null hypothesis that this pattern could have arisen by coalescent stochasticity alone. In short, the classic pattern expected from an allopolyploid origin was confirmed.

### Explanation of the minority pattern

In one gene (Fig. 1a) we could see a pattern that deviates from allopolyploidy. In this case, we found instead that the two subclades formed a monophyletic group. Such a pattern could not be attributed to stochasticity, inference error, or other effects. The length of the relevant branch in that gene tree (gene 1) spans around 2.5 coalescent units (c. 1 Ma (at c. 2 year generation time [65]) / 204,000 gene copies), which argues against deep coalescence as the cause of this topological pattern. That is, for the next closest branch in the gene tree (containing *M. sativa* and M. *sp.* alleles) to represent more closely related species to one genome in *M. arborea*/*strasseri* (which would match allopolyploidy), but more distantly related in this gene tree due to deep coalescence, requires retaining ancestral polymorphisms for a long time. This duration would need to be *at least* as long as the branch length from their divergence to the *M. arborea* clades’ common ancestor, which is around 1 Ma. This occurs c. only 10% of the time [66]. We speculate instead that a fraction of the genome may have undergone tetrasomic inheritance for a period of time that allowed the fixation of alleles from one parental genome. Subsequently, the restoration of disomic inheritance would then have allowed divergence into two sub-genomes for the genetic material including this locus. This would be consistent with a segmental allopolyploid model, but further testing is required.

The segmental allopolyploid model would also explain the second gene inconsistent with allopolyploidy, where some *M. strasseri* alleles were not grouped with the other alleles from this species and those from *M. arborea*. This could instead be due to the incomplete fixation of polymorphisms during tetrasomic inheritance.

### New data collection methods allowed the discovery of a complex evolutionary history

Earlier work using three low-copy nuclear genes on *M. arborea* showed no signs of polyploidy within this genome [33, 53]. The PCR products of two of these genes were checked for the presence of additional sequence variation using single strand conformation polymorphism [67], but no additional alleles or copies were found [33]. A third gene was cloned, but only a single clone sequence was reported [53]. Thus, neither study was able to discern the mode of origin for this polyploid species. The failure to detect additional copies in three genes suggests that reduction in copy number might be frequent in this genome, further highlighting the utility of sampling many markers. The use of a single set of PCR primers may also have been a limiting factor, as gene copies undergoing pseudogenization may not amplify as efficiently as functionally conserved copies (i.e., if the priming sites have changed). Our data for this study was gathered independently of locus-specific PCR primers, using gene capture (i.e., solution hybridization of DNA targets to RNA probes). This technique is probably more robust to sequence variation, given that successful capture relies only on an overall percentage match between target and probe across the entire probe length, rather than a specific sequence match at the priming site.

When we used unphased sequences (where a single sequence is the placeholder for all four alleles from a tetraploid) we inferred gene trees in conflict with the analyses from phased sequences. Clearly, consensus sequences in unphased analyses do not allow for the recovery of two positions for homoeologues in a single gene tree. However, it may be possible to infer both positions among several gene trees with unphased sequences, if the gene trees can each recover one of these origins correctly. What we found revealed a more serious problem. Instead of recovering one or the other homoeologous position in each gene tree (as seen in phased analyses), we saw that in most cases neither position was supported. Further, clades containing sequences from other species were often affected, with either degraded support, or supported but different relationships. These alternative relationships seen in unphased sequence analyses are undoubtedly spurious, because the fundamental assumption of tree-like relationships among terminals is violated when the sequences of allopolyploids are handled this way. We recommend that sequences constructed from the majority nucleotide at each position never be used unless the reads have been phased, when the object of an analysis is to infer polyploid origins.

Even allelic relationships in diploids might be obscured if consensus sequences were used (unless the taxon sampling is scarce and far between) where alleles from the same species are likely to be monophyletic anyway. Phasing and using a single allele, e.g., the most complete one, might be generally preferable in either case because it would at least make it possible to infer one correct position per gene tree of either an allele or a homoeologue. The examination of patterns across many gene trees would then allow species or genome relationships to be inferred.

The hybrid origin of *M. arborea* and *M. strasseri* is correlated with the most developed degree of woodiness in the genus. We also found no evidence to contradict a previous interpretation that woodiness in these species is derived from a herbaceous ancestor (see [53]; but contra [30]). However, the finding that these species share an allopolyploid origin allows a new hypothesis to be framed, namely that woodiness may be a transgressive phenotype, i.e., in this case caused by hybrid polyploidization. Among the closely related diploid species to these tetraploids, *M. cretacea*, *M. pironae*, *M. rhodopea* and *M. marina* are all described as having stems arising from a “crown” (a woody rootstock), *M. papillosa* stems arise from a woody rhizome and some members of the *M. sativa* complex also have a crown [28]. We have observed in the field that *M. prostrata s.l.* also branches from a woody rootstock. The potential for woodiness is thus widespread among closely related diploids, but not at all developed to the degree found in the tetraploids. Transgressive phenotypes associated with allopolyploidy include, for example, the long cotton fibers of massive commercial importance [68] and are of general interest to evolutionary biologists as a mechanism by which potentially adaptive traits may be formed.

## Conclusions

We found evidence that two woody perennial species of *Medicago* share an allotetraploid origin. Cytological approaches alone failed to uncover this mode of origin. On the other hand, phasing the homoeologous copies was critical to determine the origin and type of ploidy for these plants. Based on these results with *Medicago*, we expect that the inference of polyploid mode of origin will be difficult unless potentially homoeologous sequences have been phased.

## Additional files

Additional file 1: Table S1. Species used in this study along with accession numbers. *PI* and *W6* numbers are from United States Department of Agriculture (USDA) accessions. *SA* numbers are from South Australian Research and Development Institute (SARDI) accessions. *Siena* refers to an accession in the 2010 seed collection list of the Botanical Museum, University of Siena, Italy (Museo Botanico, Universita’ di Siena). GB refers to University of Gothenburg herbarium. *ENA* refers to the European Nucleotide Archive. Chromosome counts are (1) reported from Small (2011) for the species (rather than the specific sample used here), or (2) reported in Eriksson et al. (2017) and derived from living material cultivated from USDA seeds grown at the University of Gothenburg (in parenthesis). (DOCX 16 kb)

Additional file 2: Table S2. Associated information regarding the genes used in the study. (DOCX 13 kb)

Additional file 3: Table S3. Mean read depth and standard deviation for each accession, across all loci. (DOCX 45 kb)

Additional file 4: Figure S1. Phylogenetic relationship of *Medicago* based on phased alleles and majority consensus sequences, genes 1 and 2. The ultrametric trees are derived from BEAST analysis using phased alleles. The trees next to each ultrametric tree are obtained by BI using the majority consensus of unphased reads. Numbers beside branches are posterior probability values. Blue and yellow boxes represent homoeologues clades, copy 1 and copy 2, consisting of alleles from *Medicago arborea* + *M. strasseri*. Red dotted boxes highlight the differences in relationship positions between the phased tree and the majority consensus tree. (PDF 356 kb)

Additional file 5: Figure S2. Phylogenetic relationship of *Medicago* based on phased alleles and majority consensus sequences, genes 3 and 4. For details see Additional file 4: Figure S1. (PDF 596 kb)

Additional file 6: Figure S3. Phylogenetic relationship of *Medicago* based on phased alleles and majority consensus sequences, genes 5 and 6. For details see Additional file 4: Figure S1. (PDF 575 kb)

Additional file 7: Figure S4. Phylogenetic relationship of *Medicago* based on phased alleles and majority consensus sequences, genes 7 and 8. For details see Additional file 4: Figure S1. (PDF 569 kb)

Additional file 8: Figure S5. Phylogenetic relationship of *Medicago* based on phased alleles and majority consensus sequences, genes 9 and 10. For details see Additional file 4: Figure S1. (PDF 547 kb)

Additional file 9: Table S4. Gene-tree based hybridisation test results. (DOCX 40 kb)
