# Supplementary material for: Allele phasing is critical to revealing a shared allopolyploid origin of Medicago arborea and M. strasseri (Fabaceae)
Source: BMC Evol Biol. 2018 Jan 27;18:9. doi: 10.1186/s12862-018-1127-z (PMC5787288; doi:10.1186/s12862-018-1127-z)
Supplement: Supplementary file 1 — Species used in this study along with accession numbers. PI and W6 numbers are from United States Department of Agriculture (USDA) accessions. SA numbers are from South Australian Research and Development Institute (SARDI) accessions. Siena refers to an accession in the 2010 seed collection list of the Botanical Museum, University of Siena, Italy (Museo Botanico, Universita’ di Siena). GB refers to University of Gothenburg herbarium. ENA refers to the European Nucleotide Archive. Chromosome counts are (1) reported from Small (2011) for the species (rather than the specific sample used here), or (2) reported in Eriksson et al. (2017) and derived from living material cultivated from USDA seeds grown at the University of Gothenburg (in parenthesis). (DOCX 16 kb) [file 12862_2018_1127_MOESM1_ESM.docx]

**Table S1.** Species used in this study along with accession numbers.

| **Samples in this study (species name and our sample number, which also corresponds to Eriksson et al. 2017)** | **Accession #** | **ENA #** | **Chromosome number for the species from Small, 2011 (counts from Eriksson et al. 2017, from the accession listed here)** |
| --- | --- | --- | --- |
| *Medicago arborea1* | PI 368041 | ERS1353866 | 32 (32) |
| *M. arborea2* | PI 330677 | ERS1353867 | 32 |
| *M. arborea3* | PI 368172 | ERS1353868 | 32 (32) |
| *M. ciliaris* | PI 498731 | ERS719974 | 16 |
| *M. coronata* | PI 498807 | ERS1353871 | 16 |
| *M. cretacea1* | PI 631721 | ERS1353872 | 16/32 (16) |
| *M. cretacea2* | W6 33709 | ERS1353873 | 16/32 (16) |
| *M. intertexta* | Siena 385 | ERS1353874 | 16 |
| *M. italica* | PI 577295 | ERS511665 | 16 |
| *M. littoralis* | PI 537222 | ERS719977 | 16 |
| *M. marina* | PI 419391 | ERS1353875 | 16 |
| *M. medicaginoides* | W6 24116 | ERS511669 | 16 |
| *M. papillosa1* | PI 464699 | ERS1353876 | 16/32 |
| *M. papillosa2* | PI 631778 | ERS1353877 | 16/32 |
| *M. sp* | PI 577372 | ERS1353878 | 16 |
| *M. pironae2* | PI 253450 | ERS1353879 | 16 |
| *M. prostrata* | PI 577447 | ERS1353863 | 16/32 (16) |
| *M. rhodopea1* | W6 19154 | ERS1353881 | 16 |
| *M. rhodopea2* | SA 43026 | ERS1353882 | 16 |
| *M. rotata* | PI 495577 | ERS1353883 | 16 |
| *M. ruthenica* | PI 245002 | ERS1353884 | 16 |
| *M. sativa* subsp. *sativa* | PI 220598 | ERS511666 | 32 |
| *M. secundiflora* | PI 537238 | ERS1353885 | 16 |
| *M. shepardii* | PI 459134 | ERS1353886 | 16 |
| *M. strasseri* | GB-0152063 | ERS1353887 | 32 |
| *M. suffruticosa subsp. leiocarpa* | W6 4952 | ERS1353888 | 16 |
| *M. truncatula* | HM001 (HapMap reference genome) |  | 16 |

*PI* and *W6* numbers are from United States Department of Agriculture (USDA) accessions. *SA* numbers are from South Australian Research and Development Institute (SARDI) accessions. *Siena* refers to an accession in the 2010 seed collection list of the Botanical Museum, University of Siena, Italy (Museo Botanico, Universita’ di Siena). GB refers to University of Gothenburg herbarium. *ENA* refers to the European Nucleotide Archive. Chromosome counts are (1) reported from Small (2011) for the species (rather than the specific sample used here), or (2) reported in Eriksson et al. (2017) and derived from living material cultivated from USDA seeds grown at the University of Gothenburg (in parenthesis).
