# Supplementary material for: Allele phasing is critical to revealing a shared allopolyploid origin of Medicago arborea and M. strasseri (Fabaceae)
Source: BMC Evol Biol. 2018 Jan 27;18:9. doi: 10.1186/s12862-018-1127-z (PMC5787288; doi:10.1186/s12862-018-1127-z)
Supplement: Supplementary file 2 — Associated information regarding the genes used in the study. (DOCX 13 kb) [file 12862_2018_1127_MOESM2_ESM.docx]

**Table S2.** Associated information regarding the genes used in the study.

| **Gene #** | **Gene reference^1^** | **Full length of gene** | **Exon/intron (total length used)** | **Genomic block^2^** |
| --- | --- | --- | --- | --- |
| 1 | Medtr1g046550.2 | 4517bp | Exons 3-8 (3253) | C1b1 |
| 2 | Medtr2g038270.1 | 7189bp | Exons 3-9 (2422) | C2b2 |
| 3 | Medtr3g079830.1 | 4466bp | Exons 3-9 (2641) | C3b3 |
| 4 | Medtr7g088900.1 | 4450 bp | Exons 2-9 (2883) | C7b13 |
| 5 | Medtr7g088910.1 | 4519 bp | Exons 3-11 (2353) | C7b13 |
| 6 | Medtr2g122490.1 | 7924 bp | Exons 6-11 (2162) | C2b15 |
| 7 | Medtr7g005790.1 | 4051 bp | Exons 5 to end (2461) | C7b16 |
| 8 | Medtr8g025590.1 | 3065 bp | Exons 3 to end (2251) | C8b17 |
| 9 | Medtr4g080820.1 | 3315bp | All (2566) | C4b19 |
| 10 | Medtr3g113790.1 | 11465bp | Exons 18-23 (2727) | C3b20 |

^1^ *Medicago trunctula* genome version Mt.3.0

^2^ As per Sousa et al. (2014).
