# Supplementary material for: Allele phasing is critical to revealing a shared allopolyploid origin of Medicago arborea and M. strasseri (Fabaceae)
Source: BMC Evol Biol. 2018 Jan 27;18:9. doi: 10.1186/s12862-018-1127-z (PMC5787288; doi:10.1186/s12862-018-1127-z)
Supplement: Supplementary file 3 — Mean read depth and standard deviation for each accession, across all loci. (DOCX 45 kb) [file 12862_2018_1127_MOESM3_ESM.docx]

**Table S3.** Mean read depth and standard deviation for each accession, across all loci.

| **Species** | **Accession #** | **Mean Read depth (Coverage) / (Standard Deviation)** | | | | | | | | | |
| --- | --- | --- | --- | --- | --- | --- | --- | --- | --- | --- | --- |
|  |  | **Gene #** | | | | | | | | | |
|  |  | 1 | 2 | 3 | 4 | 5 | 6 | 7 | 8 | 9 | 10 |
| *Medicago arborea1* | PI368041 | 64,4 **(11,9)** | 141,6 **(77,0)** | 86,9 **(34,2)** | 126,6 **(59,5)** | 156,4 **(64,6)** | 95,7 **(41,7)** | 113,4 **(59,9)** | 138,5 **(69,9)** | 153,7 **(94,1)** | 147,5 **(75,8)** |
| *M. arborea2* | PI330677 | 59,3 **(31,5)** | 145,5 **(79,6)** | 96,9 **(43,0)** | 123,7 **(69,0)** | 167,0 **(66,8)** | 92,0 **(42,8)** | 113,2 **(63,8)** | 140,3 **(74,8)** | 163,9 **(98,1)** | 148,9 **(76,0)** |
| *M. arborea3* | PI368172 | 122,2 **(62,8)** | 150,1 **(60,8)** | 100,4 **(35,4)** | 138,5 **(52,2)** | 178,4 **(62,2)** | 120,1 **(48,2)** | 122,4 **(64,0)** | 148,8 **(60,1)** | 144,2 **(75,0)** | 171,2 **(65,1)** |
| *M. ciliaris* | PI498731 | 28,9 **(13,7)** | 32,1 **(16,6)** | 28,6 **(14,1)** | 28,7 **(14,4)** | 32,9 **(18,5)** | 27,3 **(14,9)** | 26,1 **(11,9)** | 22,3 **(11,0)** | 38,6 **(30,2)** | 28,5 **(18,0)** |
| *M. coronata* | PI498807 | 103,0 **(54,0)** | 215,0 **(107,5)** | 197,4 **(78,0)** | 199,3 **(90,5)** | 224,5 **(72,4)** | 147,4 **(67,6)** | 147,7 **(81,4)** | 124,8 **(78,9)** | 219,3 **(118,7)** | 206,2 **(101,0)** |
| *M. cretacea1* | PI631721 | 22,2 **(12,7)** | 38,3 **(17,4)** | 32,7 **(14,9)** | 40,2 **(16,6)** | 53,2 **(21,3)** | 33,4 **(17,1)** | 35,8 **(21,4)** | 40,7 **(24,5)** | 43,9 **(26,5)** | 55,9 **(32,0)** |
| *M. cretacea2* | W633709 | 30,4 **(21,5)** | 74,3 **(39,1)** | 38,5 **(30,8)** | 52,7 **(28,6)** | 69,4 **(26,8)** | 38,1 **(22,7)** | 55,3 **(33,3)** | 68,3 **(43,5)** | 62,6 **(41,1)** | 75,6 **(47,5)** |
| *M. intertexta* | Sienna | 87,9 **(38,2)** | 181,3 **(89,0)** | 173,3 **(76,5)** | 151,8 **(79,3)** | 207,3 **(81,0)** | 119,3 **(61,7)** | 143,5 **(68,0)** | 120,5 **(75,5)** | 216,7 **(121,7)** | 145,5 **(99,4)** |
| *M. italica* | PI 577295 | 60,1 **(28,9)** | 127,7 **(67.0)** | 116,8 **(43,8)** | 144,6 **(50,9)** | 161,8 **(55,8)** | 126,9 **(43,2)** | 80,0 **(43,3)** | 121,3 **(64,4)** | 139,5 **(67,5)** | 142,1 **(86,6)** |
| *M. littoralis* | PI 537222 | 63,6 **(28,2)** | 99,5 **(48,8)** | 95,8 **(41,4)** | 113,0 **(40,9)** | 118,4 **(45,7)** | 91,4 **(30,8)** | 77,2 **(49,3)** | 94,8 **(42,0)** | 102,9 **(58,6)** | 109,7 **(56,5)** |
| *M. marina* | PI 419391 | 26,2 **(14,4)** | 46,1 **(23,2)** | 28,4 **(12,7)** | 46,7 **(18,3)** | 52,1 **(22,0)** | 25,9 **(14,3)** | 37,3 **(24,9)** | 41,1 **(21,9)** | 43,0 **(32,9)** | 41,7 **(20,6)** |
| *M. medicaginoides* | W6 24116 | 83,9 **(82,3)** | 86,4 **(48,9)** | 87,5 **(32,4)** | 107,7 **(39,9)** | 78,1 **(34,4)** | 76,9 **(37,5)** | 82,9 **(38,0)** | 75,7 **(42,4)** | 115,6 **(70,5)** | 104,7 **(74,5)** |
| *M. papillosa1* | PI 464699 | 17,4 **(8,8)** | 37,8 **(14,7)** | 30,1 **(17,8)** | 27,9 **(13,3)** | 37,1 **(14,6)** | 25,8 **(9,1)** | 26,0 **(16,5)** | 33,5 **(15,2)** | 35,9 **(21,7)** | 45,7 **(21,7)** |
| *M. papillosa2* | PI 631778 | 22,6 **(14,4)** | 48,2 **(22,2)** | 40,2 **(20,3)** | 38,1 **(15,3)** | 48,5 **(20,2)** | 37,8 **(21,4)** | 39,1 **(24,0)** | 35,2 **(18,8)** | 44,9 **(30,9)** | 56,2 **(27,1)** |
| *M. sp* | PI 577372 | 14,9 **(10,7)** | 39,2 **(20,6)** | 31,3 **(14,2)** | 33,5 **(17,6)** | 51,5 **(17,2)** | 24,3 **(14,1)** | 33,5 **(22,1)** | 37,4 **(19,0)** | 45,3 **(24,8)** | 41,7 **(23,3)** |
| *M. pironae2* | PI 253450 | 44,5 **(22,9)** | 75,1 **(29,4)** | 48,7 **(20,2)** | 62,5 **(22,6)** | 62,7 **(19,9)** | 56,6 **(20,9)** | 54,5 **(28,3)** | 66,8 **(20,4)** | 62,2 **(32,7)** | 74,4 **(29,1)** |
| *M. prostrata* | PI 577447 | 97,5 **(52,5)** | 143,8 **(59,5)** | 104,6 **(38,0)** | 118,9 **(48,3)** | 143,9 **(47,4)** | 115,3 **(54,2)** | 104,6 **(53,8)** | 143,2 **(56,9)** | 119,7 **(60,7)** | 158,8 **(71,2)** |
| *M. rhodopea1* | W6 19154 | 21,8 **(15,4)** | 48,0 **(24,1)** | 35,6 **(18,3)** | 42,3 **(21,1)** | 60,1 **(22,7)** | 30,0 **(13,8)** | 39,5 **(26,0)** | 42,2 **(20,7)** | 55,5 **(33,0)** | 57,2 **(30,8)** |
| *M. rhodopea2* | SA 43026 | 32,4 **(21,1)** | 79,1 **(33,8)** | 53,3 **(21,1)** | 54,8 **(20,6)** | 70,7 **(22,3)** | 59,3 **(26,0)** | 57,6 **(28,8)** | 67,8 **(24,6)** | 77,6 **(44,5)** | 82,4 **(34,6)** |
| *M. rotata* | PI 495577 | 41,7 **(26,3)** | 75,7 **(40,8)** | 57,7 **(25,8)** | 59,9 **(25,3)** | 58,2 **(27,2)** | 47,0 **(28,4)** | 37,4 **(28,4)** | 59,8 **(40,8)** | 79,9 **(50,7)** | 74,9 **(49,9)** |
| *M. ruthenica* | PI 245002 | 38,8 **(27,8)** | 75,5 **(43,5)** | 66,2 **(35,3)** | 78,5 **(31,4)** | 76,1 **(39,0)** | 56,2 **(26,0)** | 51,5 **(33,6)** | 62,1 **(40,2)** | 98,7 **(66,4)** | 101,1 **(65,6)** |
| *M. sativa subsp. sativa* | PI 220598 | 42,0 **(26,8)** | 78,7 **(39,5)** | 57,7 **(31,6)** | 63,0 **(34,5)** | 77,9 **(30,3)** | 58,7 **(26,5)** | 56,3 **(36,0)** | 72,3 **(36,4)** | 68,2 **(47,6)** | 87,1 **(53,6)** |
| *M. secundiflora* | PI 537238 | 52,0 **(31,5)** | 92,2 **(47,6)** | 87,2 **(44,1)** | 102 **(43,5)** | 114,1 **(43,4)** | 66,7 **(33,0)** | 77,6 **(60,7)** | 75,8 **(40,4)** | 103,2 **(70,6)** | 93,9 **(55,3)** |
| *M. shepardii* | PI 459134 | 21,7 **(14,6)** | 49,5 **(25,8)** | 38,2 **(21,3)** | 40,3 **(20,4)** | 46,6 **(20,1)** | 32,0 **(17,0)** | 58,0 **(32,6)** | 33,4 **(22,0)** | 41,6 **(30,3)** | 48,3 **(29,2)** |
| *M. strasseri* | G. Pediados, Iraclio | 52,7 **(25,5)** | 84,4 **(47,4)** | 74,8 **(34,7)** | 70,0 **(31,3)** | 93,2 **(38,5)** | 68,8 **(37,4)** | 68,3 **(43,6)** | 83,6 **(44,2)** | 86,2 **(64,0)** | 94,1 **(48,5)** |
| *M. suffruticosa subsp. leiocarpa* | W6 4952 | 39,5 **(23,4)** | 46,8 **(33,6)** | 43,0 **(18,3)** | 50,2 **(22,0)** | 386,6 **(633,4)** | 43,3 **(21,2)** | 43,1 **(22,1)** | 29,6 **(19,6)** | 67,4 **(36,4)** | 61,1 **(35,4)** |
