# Supplementary figures and images for: Allele phasing is critical to revealing a shared allopolyploid origin of Medicago arborea and M. strasseri (Fabaceae)
Source: BMC Evol Biol. 2018 Jan 27;18:9. doi: 10.1186/s12862-018-1127-z (PMC5787288; doi:10.1186/s12862-018-1127-z)

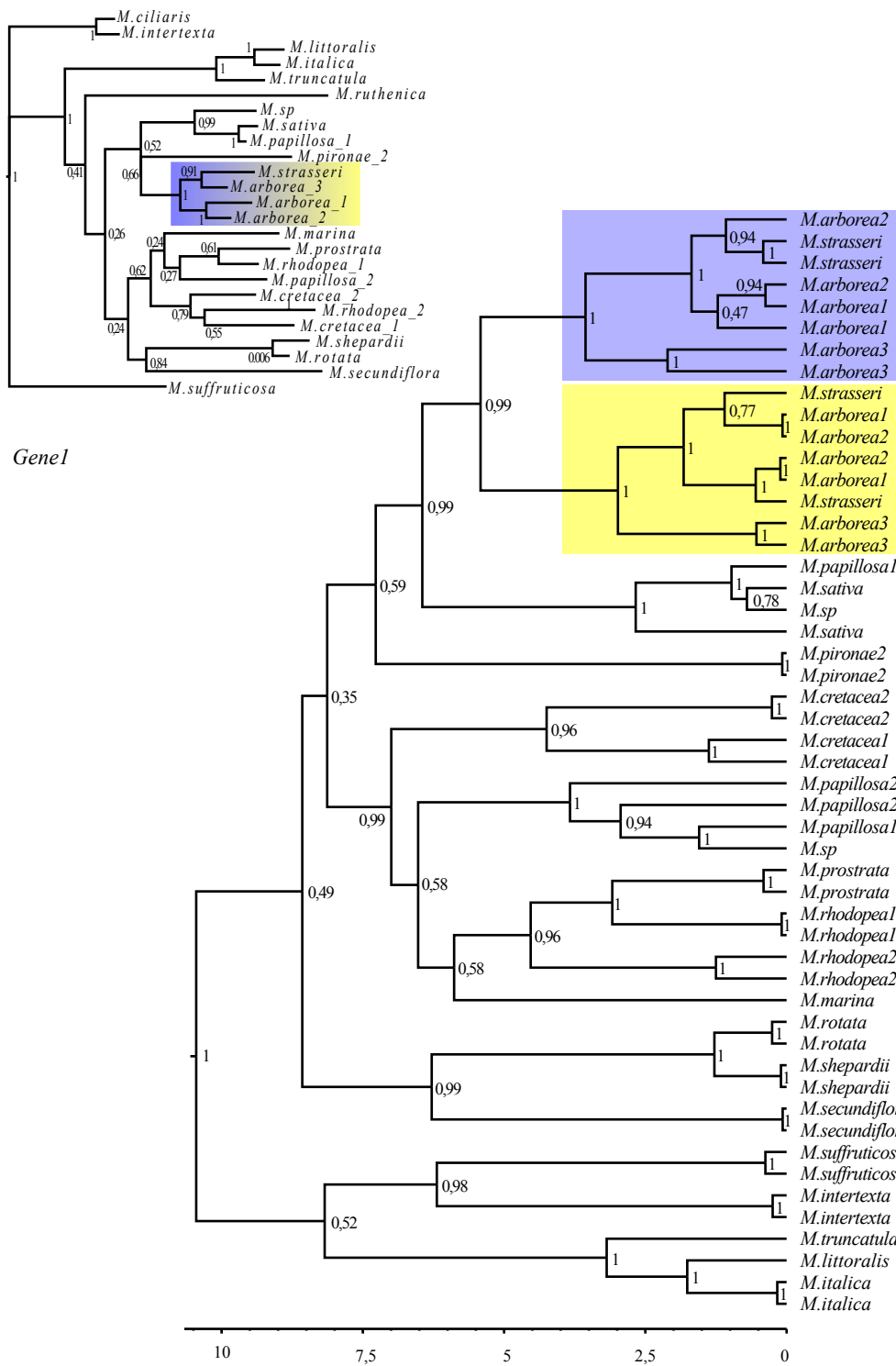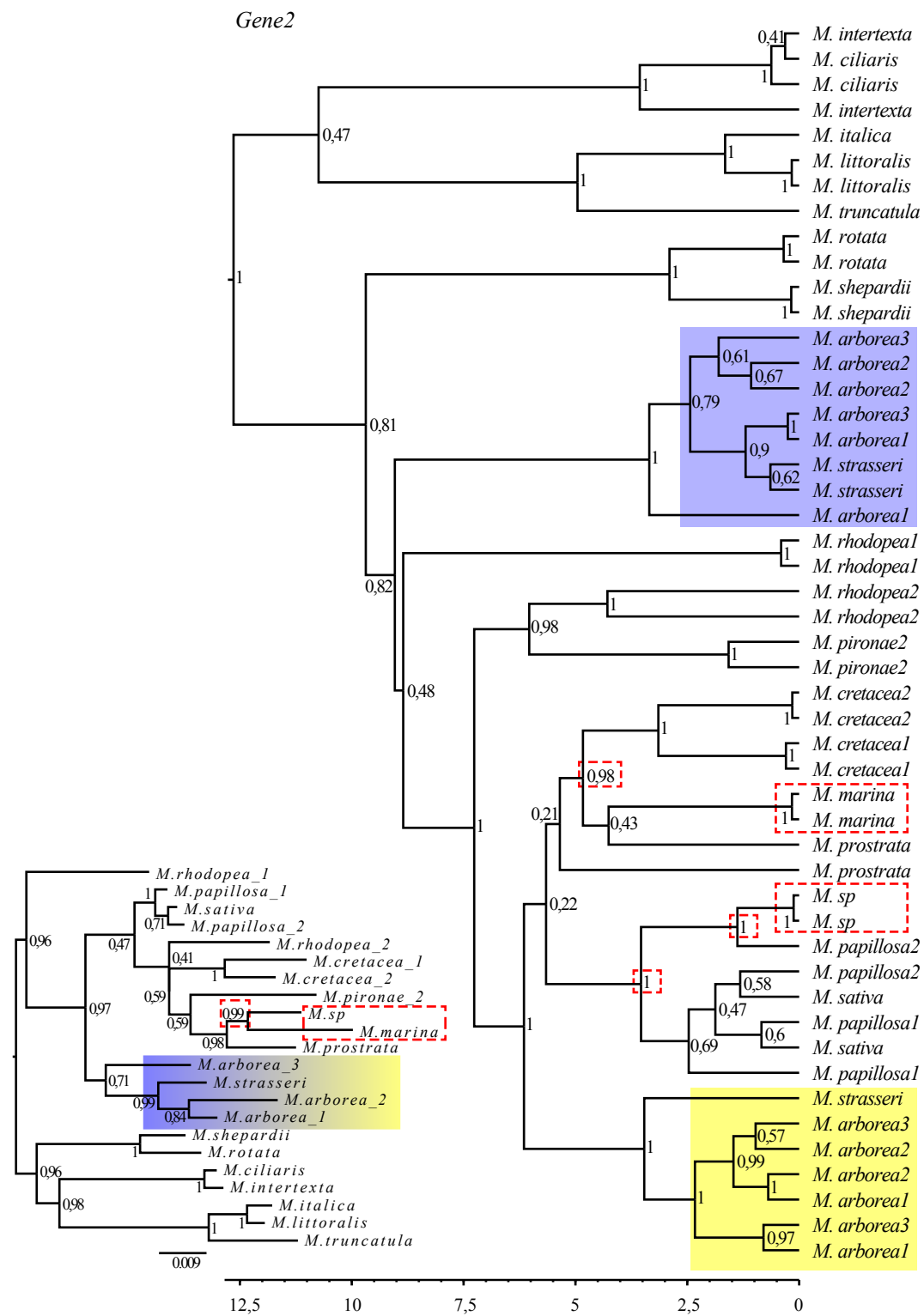

Supplement: Supplementary file 4 — Phylogenetic relationship of Medicago based on phased alleles and majority consensus sequences, genes 1 and 2. The ultrametric trees are derived from BEAST analysis using phased alleles. The trees next to each ultrametric tree are obtained by BI using the majority consensus of unphased reads. Numbers beside branches are posterior probability values. Blue and yellow boxes represent homoeologues clades, copy 1 and copy 2, consisting of alleles from Medicago arborea + M. strasseri. Red dotted boxes highlight the differences in relationship positions between the phased tree and the majority consensus tree. (PDF 356 kb) [file 12862_2018_1127_MOESM4_ESM.pdf]

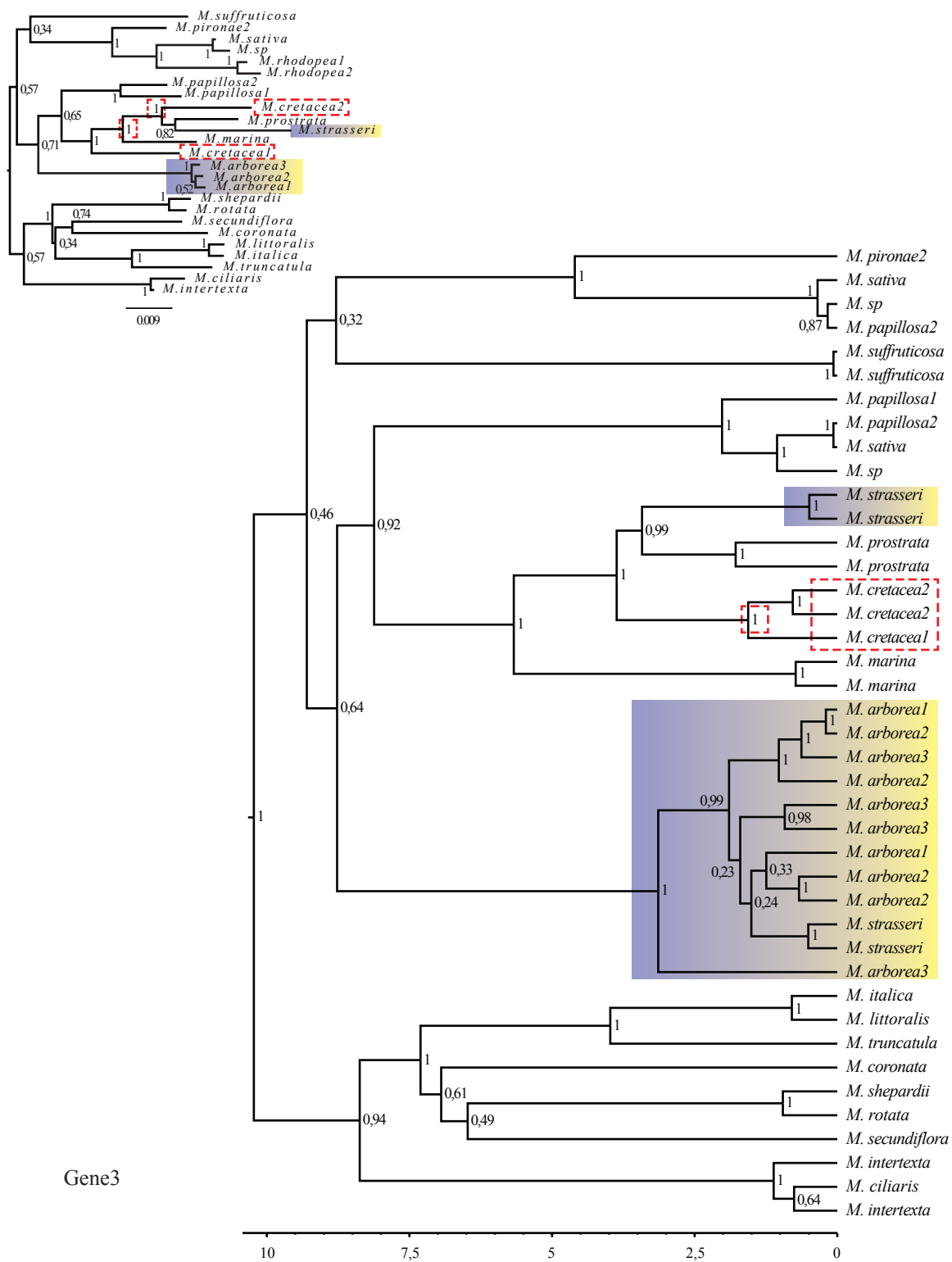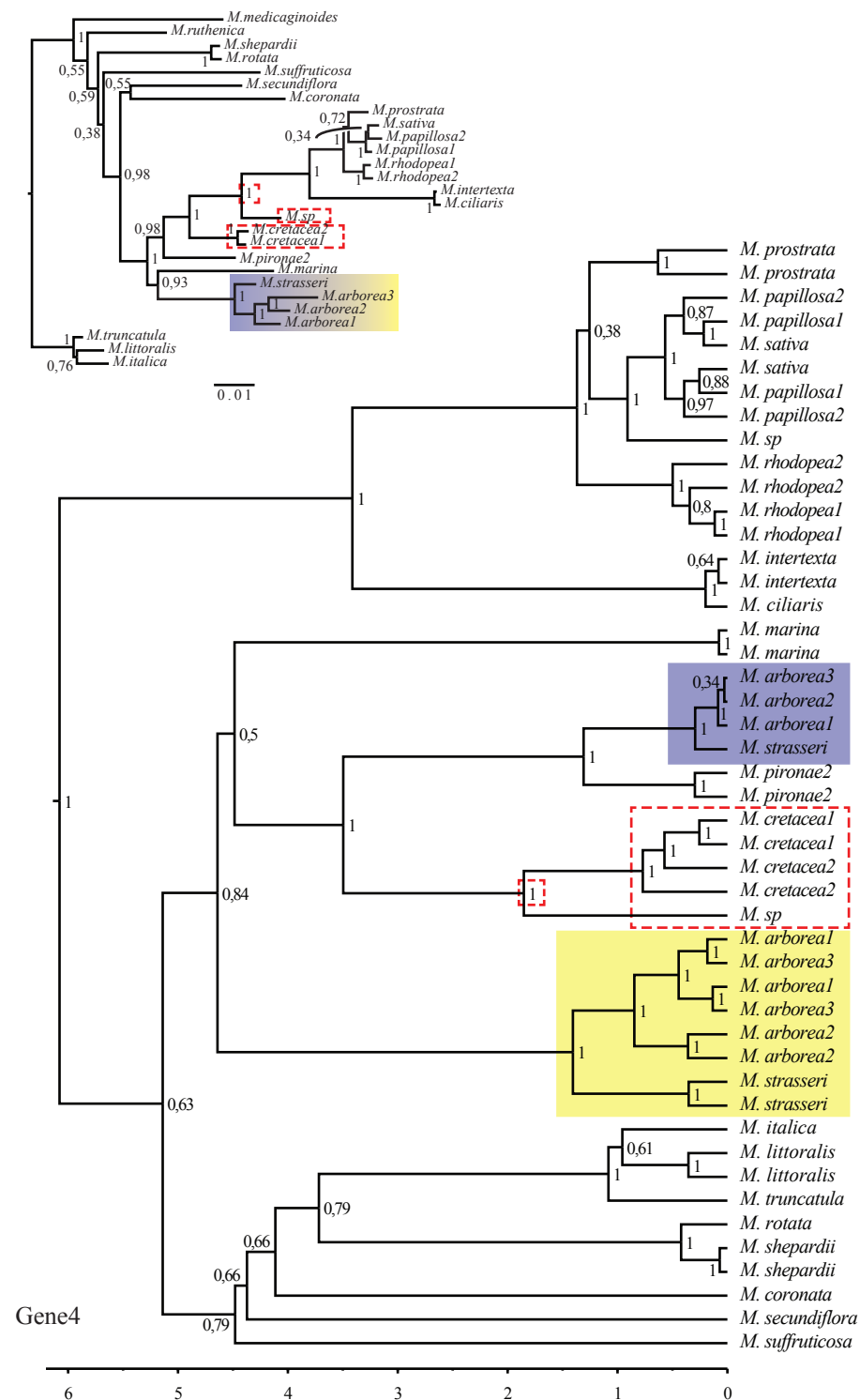

Supplement: Supplementary file 5 — Phylogenetic relationship of Medicago based on phased alleles and majority consensus sequences, genes 3 and 4. For details see Additional file 4: Figure S1. (PDF 596 kb) [file 12862_2018_1127_MOESM5_ESM.pdf]

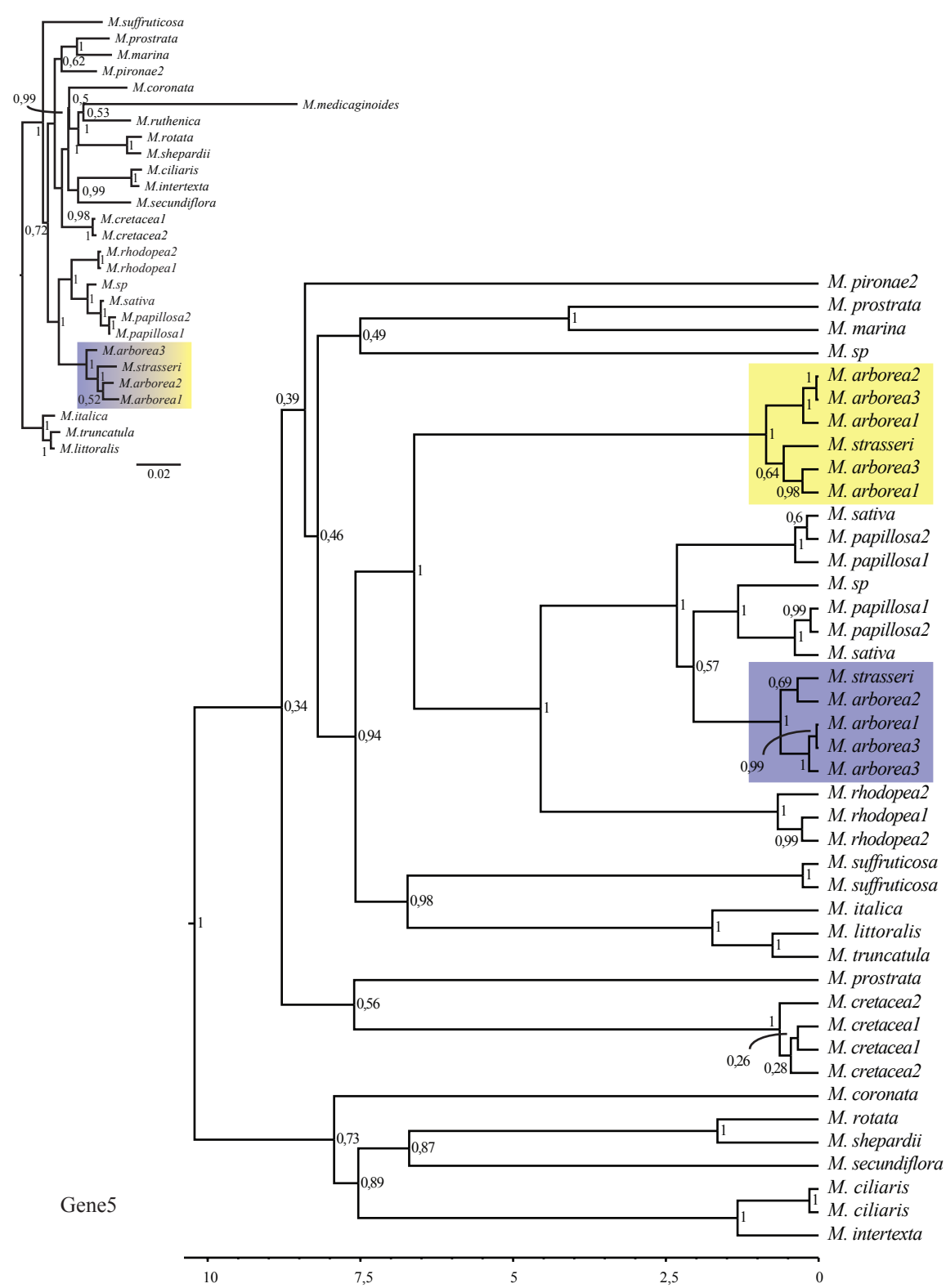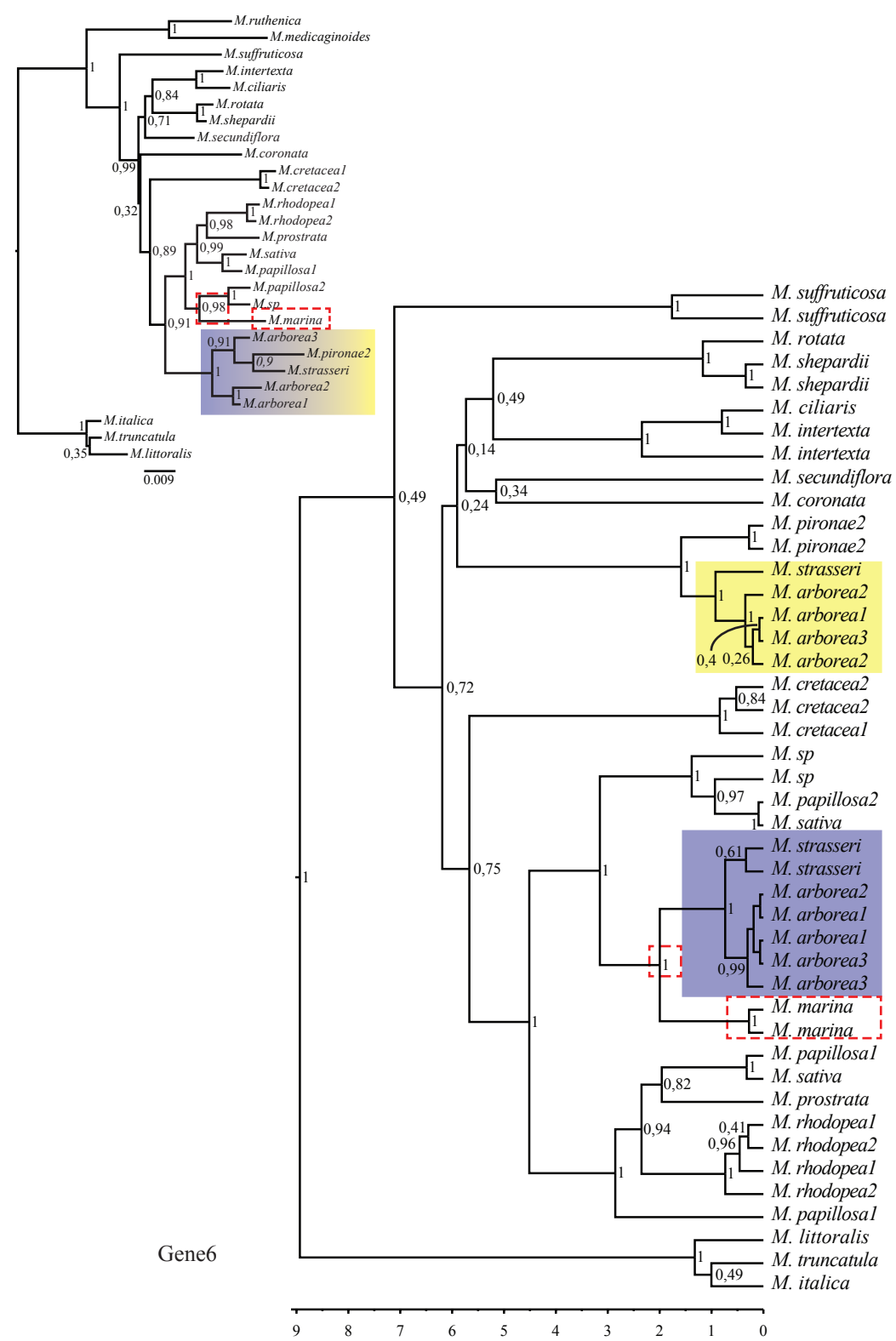

Supplement: Supplementary file 6 — Phylogenetic relationship of Medicago based on phased alleles and majority consensus sequences, genes 5 and 6. For details see Additional file 4: Figure S1. (PDF 575 kb) [file 12862_2018_1127_MOESM6_ESM.pdf]

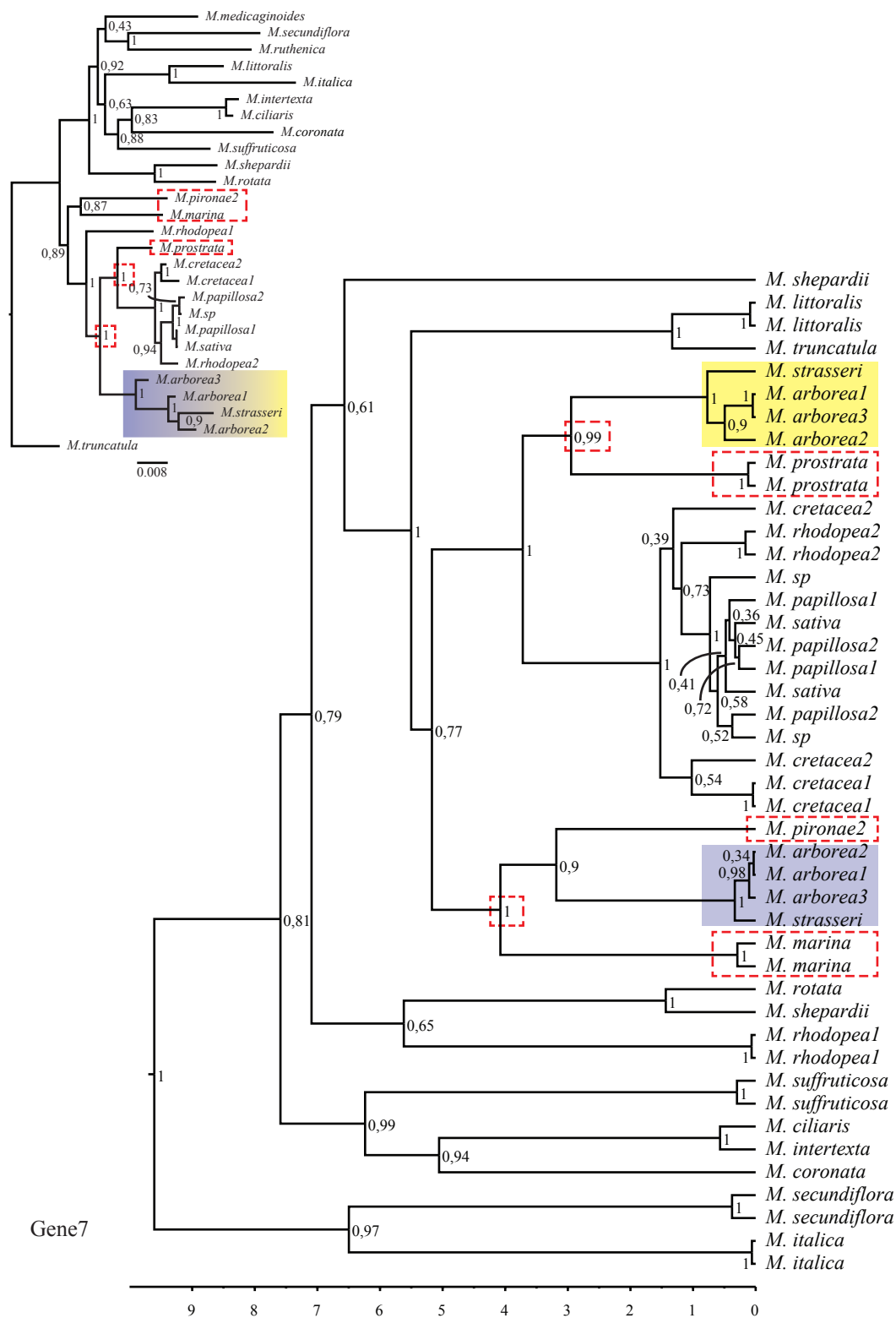

**Gene8**

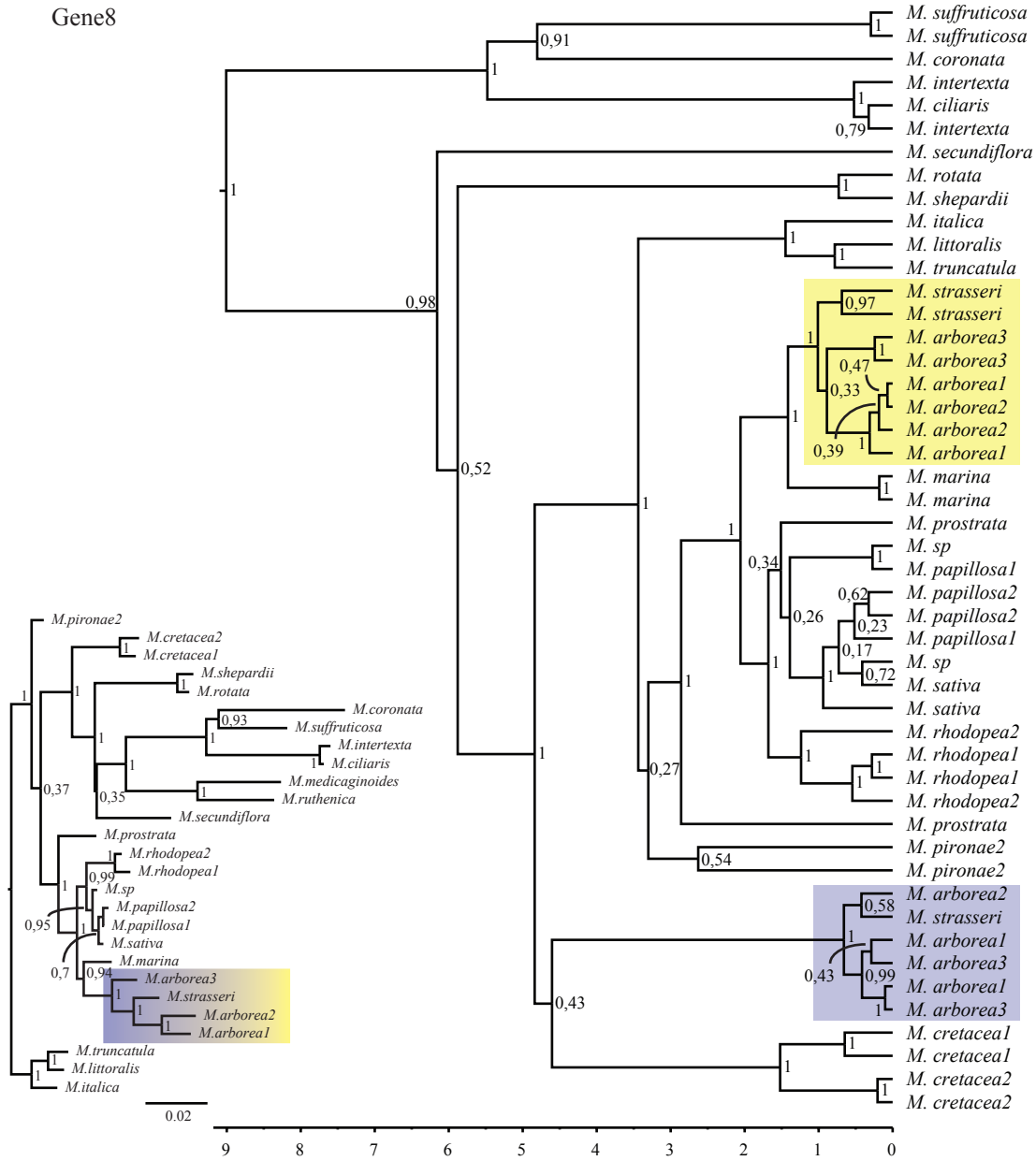

Supplement: Supplementary file 7 — Phylogenetic relationship of Medicago based on phased alleles and majority consensus sequences, genes 7 and 8. For details see Additional file 4: Figure S1. (PDF 569 kb) [file 12862_2018_1127_MOESM7_ESM.pdf]

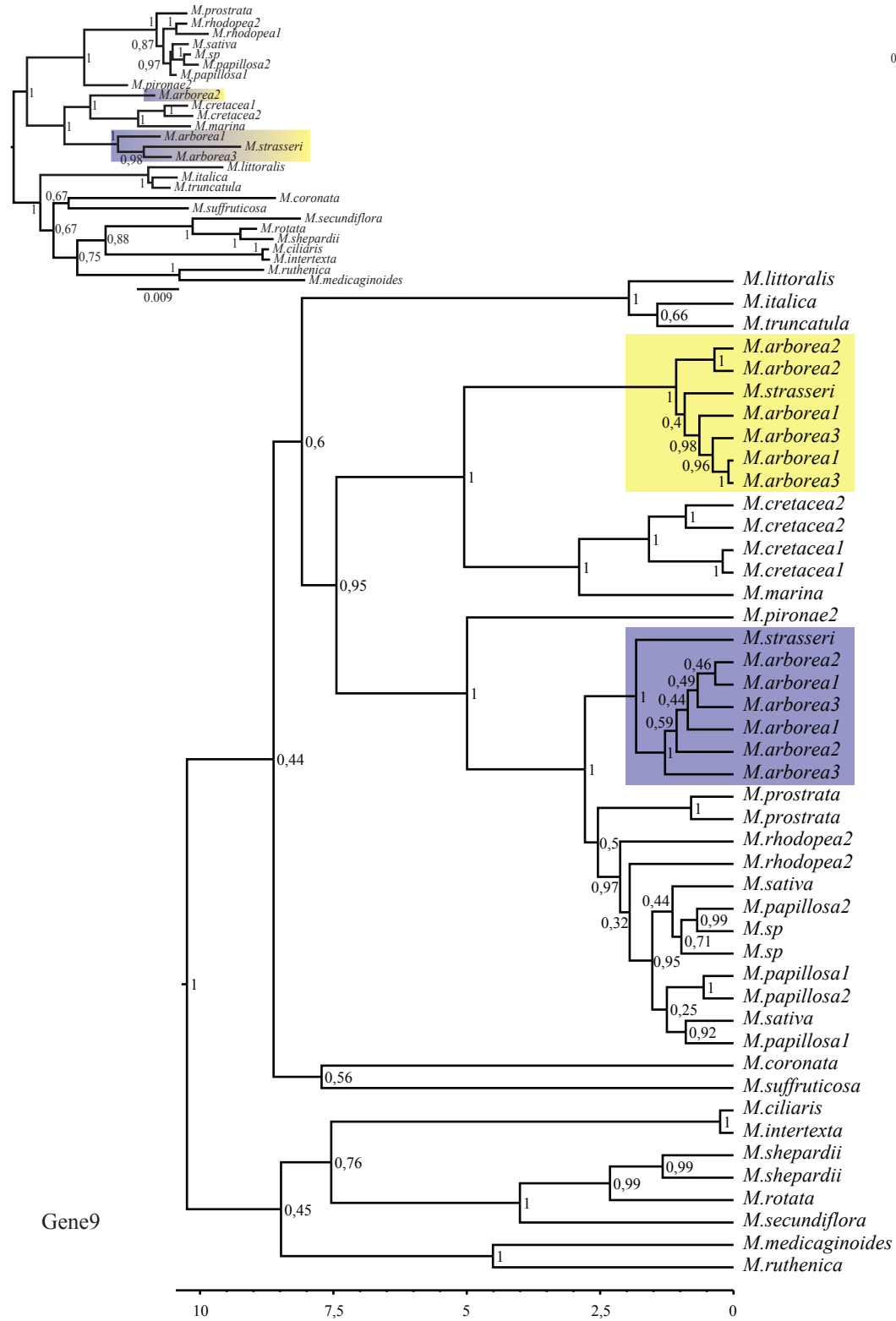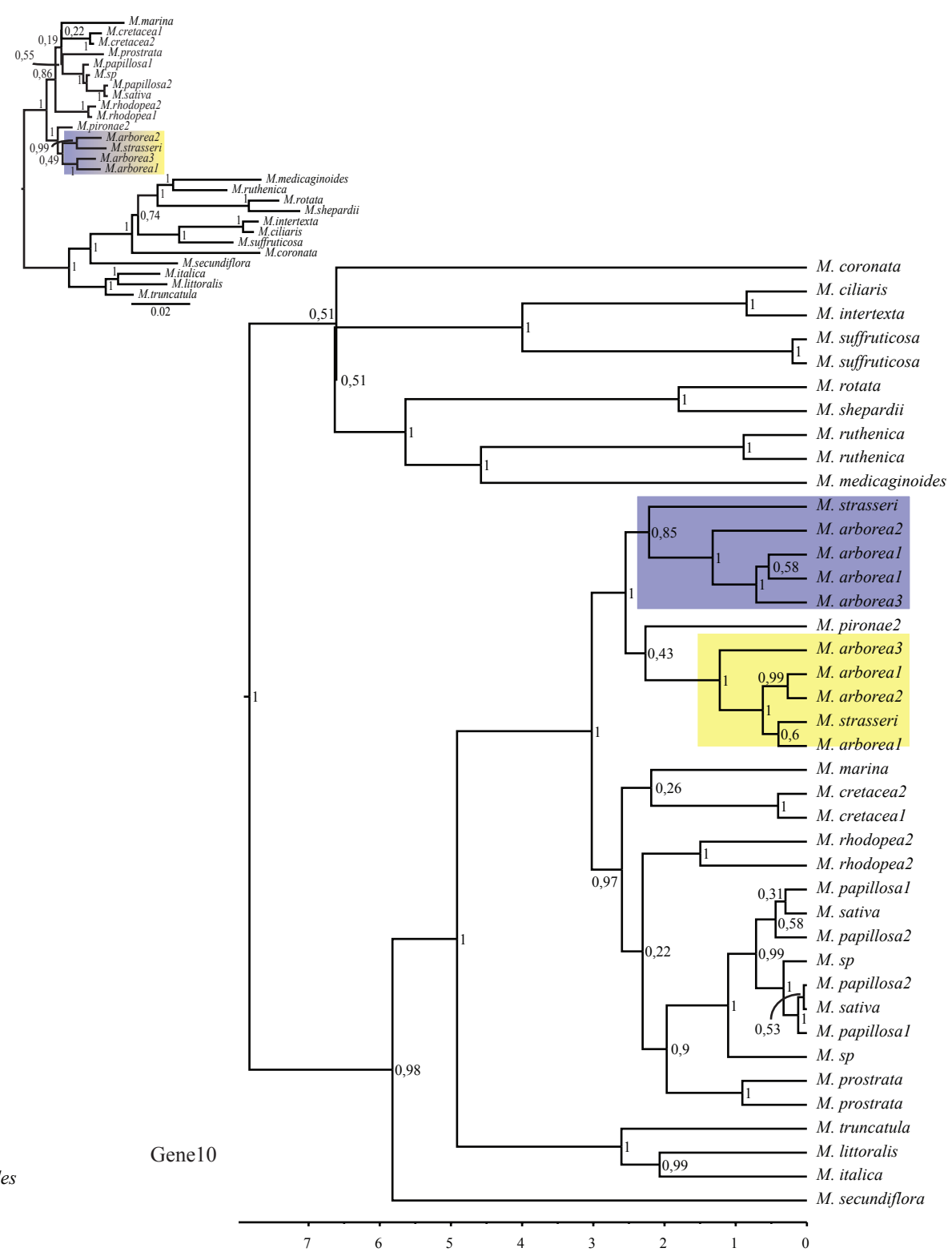

Supplement: Supplementary file 8 — Phylogenetic relationship of Medicago based on phased alleles and majority consensus sequences, genes 9 and 10. For details see Additional file 4: Figure S1. (PDF 547 kb) [file 12862_2018_1127_MOESM8_ESM.pdf]
