# Supplementary material for: Allele phasing is critical to revealing a shared allopolyploid origin of Medicago arborea and M. strasseri (Fabaceae)
Source: BMC Evol Biol. 2018 Jan 27;18:9. doi: 10.1186/s12862-018-1127-z (PMC5787288; doi:10.1186/s12862-018-1127-z)
Supplement: Supplementary file 9 — Gene-tree based hybridisation test results. (DOCX 40 kb) [file 12862_2018_1127_MOESM9_ESM.docx]

**Table S4.** Gene-tree based hybridisation test results.

|  | 107 | 131 | 132 | 137 | 141 | 142 | 147 | 148 |
| --- | --- | --- | --- | --- | --- | --- | --- | --- |
| Coalescence null 65% value^1^ | 2 | 2 | 0 | 2 | 2 | 2 | 0 | 4 |
| Observed 2.5% lower value^2^ | 4 | 2 | 6 | 10 | 10 | 4 | 4 | 0 |
| Null hypothesis | Reject | n.s. | Reject | Reject | Reject | Reject | Reject | n.s. |

^1^ Tree-to-tree distances (Robinson-Fould) between the gene trees from the posterior sample (observed trees) and the simulated set of trees under a coalescent null model (ILS only).

^2^ Tree-to-tree distances (Robinson-Fould) between the gene trees from the posterior sample (observed trees) containing one position of *M. arborea* (the other removed) and those trees from the posterior sample containing the other position of *M. arborea* (the first removed).
